# The impact of cognitive schema on learning transfer ability and stability of classical Chinese poetry

**DOI:** 10.1371/journal.pone.0336135

**Published:** 2025-11-06

**Authors:** Dawei Liu, Ping He, Huifen Yan

**Affiliations:** 1 College of Teacher Education, Hubei University of Education, Wuhan, China; 2 Undergraduate School, National University of Defense Technology, Changsha, China; 3 Institute of Educational Sciences, Wuhan University, Wuhan, China; Bahir Dar University, ETHIOPIA

## Abstract

Classical Chinese poetry is a condensed vessel of Chinese culture, bearing the core ethos of history, philosophy, and ethics. The symbolic imagery system in poetry facilitates the construction of cognitive schemata, thereby advancing transfer learning. Based on Schema Theory and Cognitive Structure Migration Theory, this research conducted a controlled experiment, with the participants being undergraduate students, to examine how “cognitive schema’‘ influences transfer ability and stability in learning classical Chinese poetry. The results revealed that the experimental group (n = 63) achieved higher scores in both tests, indicating that learners who employed the cognitive schema learning method demonstrated greater transfer ability and stability than the control group (n = 64). Significant differences in results between the experimental group and control group highlighted the positive impact of cognitive schema on learners’ transfer ability and stability in learning classical Chinese poetry. It is hoped that this research will provide valuable implications for the teaching and learning of classical Chinese poetry.

## 1. Introduction

China has a rich poetic tradition. As a nation with deep-rooted poetic traditions, classical Chinese poetry embodies a cultural continuum that spans millennia and serves dual pedagogical functions in contemporary education. This duality manifests in two interconnected roles: first, as the primary vehicle for safeguarding and transmitting China’s intangible cultural heritage; and second, as a cornerstone of the national language curriculum. In recent years, the importance of the Chinese subject has steadily increased due to the development and reform of China’s education. According to the “Chinese Curriculum Standards for Compulsory Education’‘ (2011 edition) [1], it is explicitly mandated that students should complete the study of at least 240 canonical poems during the nine-year compulsory education phase. Additionally, students are encouraged to enhance their abilities of appreciation and aesthetic sensibilities through the accumulation, perception, and application of these poems. Since 2017, all primary and secondary schools in China have adopted new textbooks, resulting in a significant increase of the number of traditional subjects, including classical Chinese poetry across all grades levels. Consequently, this development calls for greater attention to and exploration of systematic and effective methods for teaching and learning classical Chinese poetry.

A variety of scholars have proposed different approaches to learning classical Chinese poetry in recent years, including “contextual priming’‘ [2,3], “integration of poetry and painting’‘ [4,5], “recreation of poetic conception’‘ [6], and “imagery association’‘ [7,8]. However, most of the published articles on this subject primarily illustrate authors’ perspectives based on individual teaching experiences, with a greater emphasis on how students memorize classical Chinese poetry. There is a significant lack of empirical studies focused on how to enhance transfer ability and transfer stability in learning classical Chinese poetry.

The transfer ability and transfer stability of learning ancient Chinese poetry are dialectical related, complementing and coordinating each other’s development. Transfer ability is the premise and foundation of stability. On the other hand, The process of achieving transfer stability is precisely the process of deepening and internalizing transfer ability, which in turn promotes broader and more advanced transfer abilities. Transfer ability and stability are closely related to the ultimate goal of learning classical Chinese poetry, namely, its application and transfer. By gaining a profound understanding of social issues, human emotions, and the moral principles expressed by poets, students should be empowered not only to promote national culture but also to enhance their own aesthetic sensibilities. This process fosters patriotism and sentiments such as homesickness and friendship, improves their interpersonal skills, advances dialectical thinking, and helps them develop accurate perspectives on life and values in a subtle manner.

Therefore, this study employs an quasi-experimental method to examine the impact of cognitive schema on transfer ability and stability in the learning of classical Chinese poetry, grounded in Cognitive Structure Migration Theory [9] and Schema Theory [10]. It is hoped that this study can provide insights into the theoretical building of poetry schema, facilitate the earning of classical Chinese poetry, and cultivate students’ comprehensive literacy. The following research questions will be addressed: 1) Does the cognitive schema approach to learning classical Chinese poems facilitate greater transfer of learning? 2) In what way does the cognitive schema method influence the stability of transfer in learning classical Chinese poems?

## 2. Cognitive structure migration theory and schema theory

### 2.1 Cognitive structure migration theory

Psychologists Mark K. Singley and John R. Anderson [11] stated in their book *The Transfer of Cognitive Skills*: “No field is more suitable for testing learning theories than transfer’‘ (p.5). As early as 1913, Thorndike proposed that learning transfer involves the transfer of identical connections [12]. Progress in one type of learning can only be transferred to another when there are common connections between the two types. The Gestalt school posited that learning transfer is driven by similar functions and involves understanding the relationships among the components of the entire situation [13]. Based on the notion that knowledge acquisition involves the formation of specific cognitive structures in the mind, which are constructed through encoding systems, Bruner [14] defined transfer as the process of using an acquired encoding system to learn new knowledge. Ausubel [15] believed that learning new material occurs based on the learner’s existing cognitive structure, asserting that the learning process of new material necessarily includes transfer. The transfer of learning is the process of “establishing ‘nonarbitrary and substantive (nonverbatim) connections’ (i.e., assimilation) between new material and appropriate existing relevant ideas in the learner’s cognitive structure’‘ [16]. Cognitive understanding accumulated through previous learning can influence new learning by associating it with relevant characteristics of the existing cognitive structure.

Cognitive structure denotes an individual’s internal knowledge framework or encoding system of the real world, serving as a mental framework for perceiving, processing external information, and engaging in reasoning activities. The characteristics of an individual’s cognitive structure in terms of content and organization are referred to as cognitive structure variables, which primarily include availability, discriminability, and stability [9,15–17]. Availability denotes the extent to which relevant concepts within the learner’s existing cognitive structure can be employed to assimilate new information during the learning process. Discriminability indicates the learner’s ability to differentiate between new and old knowledge based on their current cognitive structure. Stability reflects the degree to which concepts within the learner’s cognitive structure are consolidated, playing a fixed and assimilative role in the acquisition of new information. These three cognitive structure variables are critical factors that influence the effectiveness of learning and transfer. Transfer can be categorized as positive (facilitative) or negative (interferential) based on its quality and outcomes[9]. Consequently, advance organizers can be employed to modify cognitive structure variables, thereby bridging the gap between the learner’s existing cognitive structure and new information, and facilitating positive transfer through “ideational scaffolding’‘ [14,16].

In summary, the learning process involves the transfer of information, during which the learner encodes the learning materials. This encoding creates a high-speed channel between their existing knowledge and new information, allowing for a more efficient ‘connection’ with the new material. As a result, this process enhances the availability, discriminability, and stability of the learner’s cognitive structure, and facilitates the transfer of new learning and its retention. Therefore, in teaching, the presentation of learning materials should be specifically designed to align with the variables of students’ existing cognitive structures to promote positive learning transfer. To put into this theory into the teaching practices of ancient Chinese poetry, various instructional strategies are important to be designed to help students form a large number of well-organized schemas, which can facilitate transfer ability and stability. This is because schemas are often regarded as the organizational units within cognitive structures.

### 2.2 Schema theory

Schemas are cognitive structures or knowledge frameworks that reside in memory and represent abstractions of objects in a learner’s life [18]. Bartlett [10] found in his study that the participants tended to utilize familiar schemas to comprehend new information, and these schemas further influenced their reconstruction of the original text during subsequent recall. He defined schemas as “an active organisation of past reactions, or of past experiences’‘ (p.201). Piaget [19] defined schemas as “structures or organizations of action that result from repeated experiences in similar situations and serve as a basis for transfer or generalization’‘, demonstrating their essential role in in cognitive processes such as thinking and creativity.

Schemas are stored in the brain and are derived from past experiences, serving as a form of generalized understanding of recurrent situations. They consist not only of organized knowledge but also perceptual elements [20]. Automation of both schemas and rules does not require the expenditure of additional resources, thereby compensating for the limited capacity of an individual’s working memory and reducing cognitive load [18]. This enhancement improves transfer ability and stability. Therefore, reinforcing the construction of knowledge schemas in teaching can help learners establish effective cognitive structures and facilitate the learning of new material [21,22].

In classical Chinese poetry composition, the expression of emotions is the primary focus. Poets frequently employ external entities (e.g., natural imagery) to convey emotions through figurative techniques such as analogy (“Bi’‘) and association (“Xing’‘). Consequently, when learners appreciate classical Chinese poems, they can empathize with the poetic themes and enhance their relevant cognitive load by “immersing’‘ themselves in the experience, and connecting poems to their existing life experiences and knowledge [23]. This process fosters cognitive assimilation [24] and promotes the transfer of cognitive structures [25]. By evoking emotions through the vivid descriptions of scenes and objects in classical Chinese poetry and realizing the interaction between them and the author’s emotions, learners can grasp the overall themes of the poems and successfully transfer their understanding of classical Chinese poetry. Additionally, schemas also contribute to the stability of cognitive structures, as they represent a relatively stable cognitive structure within the encoding system.

**Fig 1:**
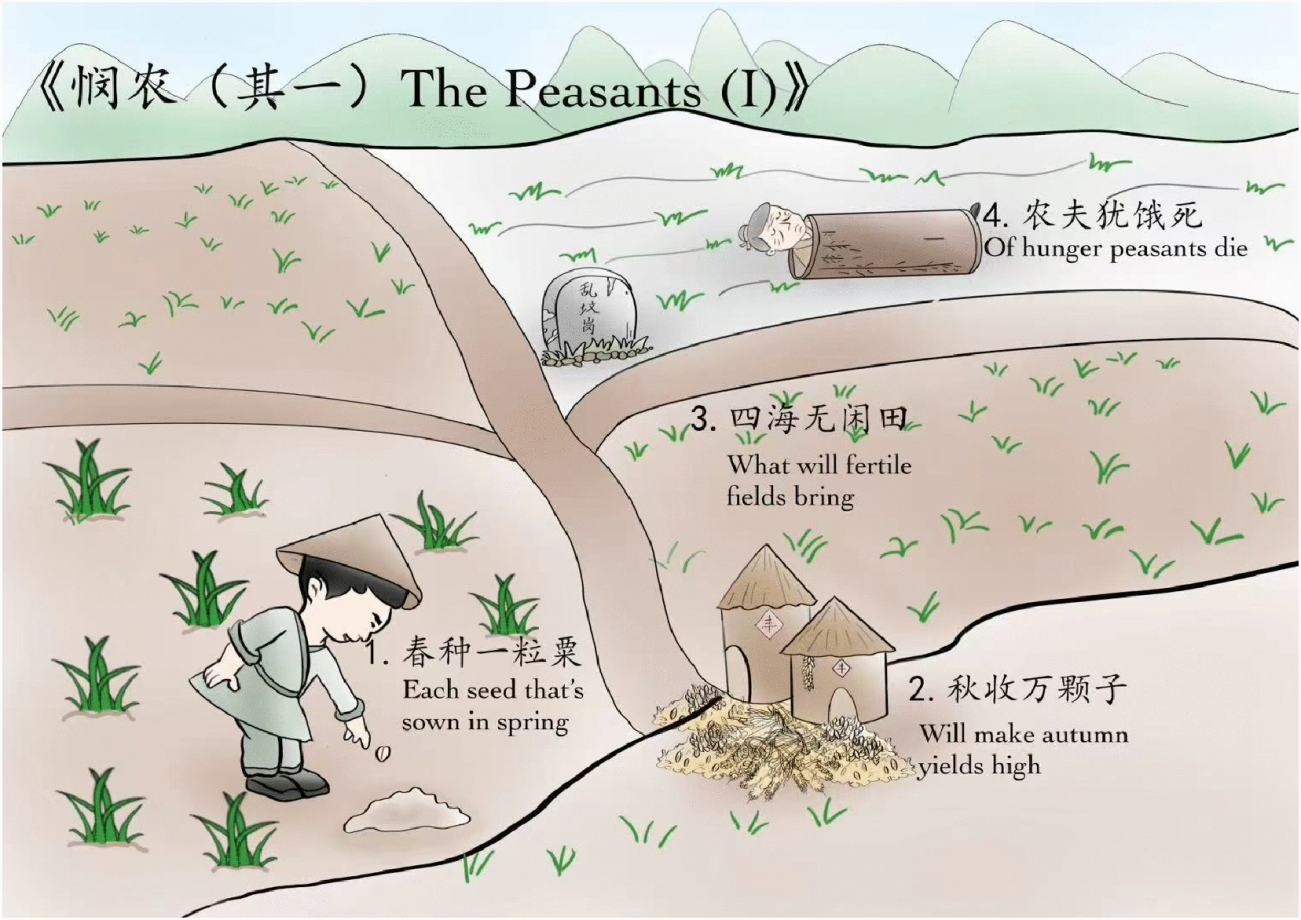
Poetry schema example.

By applying schema theory, even novice learners of classical Chinese poetry can effectively establish meaningful connections with the content of the poems. For instance, when analyzing the poem “The Peasants (I)’‘, learners can grasp both the intellectual and emotional nuances of its central theme through interpretation of representative imagery—a cultivated field depicted in the opening lines. This process enables learners to activate prior knowledge, facilitating schema activation [25] and fostering deeper engagement with the poem’s sociocultural context. Imagine a scene: the vibrant green of newly sprouted plants symbolize ‘spring’, while a farmer sowing a seed in the field represents ‘Each seed that’s sown in spring’. The golden grains symbolize ‘autumn’, while two granaries next to the field freighted with tens of thousands of golden grains foretell ‘Will make autumn yields high’. The four cultivated fields, all lush with green crops, embody ‘What will fertile fields bring’. Yet the farmer who died of starvation and lay in a straw mat is casually abandoned in an unmarked common grave, presenting ‘Of hunger peasants die’ (see Fig 1). This method has been discussed in previous study; please refer to our earlier research for further details [26]. Through this approach, learners can not only experience the emotions conveyed in the poem but also gain a deeper understanding of its message regarding diligence, thrift, sympathy and criticism. Additionally, through the imaginative representation of the image combined with the poem title “The Peasants’‘ as well as the existing knowledge about another familiar verse line, “Who knows that the meals in our plates? Every morsel of them comes with toils and pains’‘, learners can gain a rough idea of the main themes of this poem, namely the arduous lives of peasants at the time, sympathy for the peasants. This method effectively enhances learners’ understanding of the poem’s overall artistic conception and helps them connect with its content on both emotional and cognitive levels. By integrating specific scenes from the poem with schemas, learners can more easily grasp and retain the themes and meanings presented. This learning method, which incorporates the use of schemas, not only reduces cognitive load but also improves the transfer ability and stability of learning materials, enabling learners to absorb and utilize new knowledge more effectively.

Through such kind of learning method, students can memorize the poem for a longer period and better understand the implicit meaning and gist of this poem. In such a way, the transfer ability and stability of learning ancient Chinese poetry can be enhanced.

## 3. Research methods

Initially, it should be noted that the authors obtained oral consent from all the participants. Before the experiment, participants were informed of their willingness to participate and were made aware that they could withdraw at any time. Their personal information would remain confidential, and all data would be analyzed anonymously, solely for academic research purposes. This procedure was approved by the IRB. All methods were conducted in accordance with relevant guidelines and regulations, and the processes of this study have been approved by Brain Science and Learning Science Committee of Hubei Teachers Education Association. The ethical approval has been obtained from the Brain Science and Learning Science Committee of Hubei Teachers Education Association (No. S2023-027-2). The start of the recruitment period is May 9, 2023, and the end of the recruitment period is June 30, 2023.

This study employed a quasi-experimental design, which included both a control group and an experimental group. Students in the experimental group utilized the cognitive schema-based mnemonic method during the test, while those in the control group did not employ this method. Consequently, we were able to compare the learning transfer ability and stability of classical Chinese poetry between the two groups. It could also provide evidence for the effectiveness and efficiency of the new method we proposed.

### 3.1 Research participants

The present study selected undergraduate students from five classes at a normal university in Wuhan (a central city in China) as research participants, including three freshmen classes and two senior classes, which were characterized by interdisciplinary feature. Given that the freshmen had just completed the college entrance examination and possessed a foundation knowledge of classical Chinese poetry through reciting poems and extensive transfer training in high school, they were designated as the control group. The senior students, who had relatively poor transfer ability of learning classical Chinese poetry due to their focus on discipline specific coursework and lack of further transfer training after their enrollment, were assigned to the experimental group. Neither groups had received any training in classical Chinese poetry schema prior to participating in this experiment. The control group (n = 64) consisted of 40 female students and 24 male students, with an average age of 19 years; the experimental group (n = 63) comprised 55 female students and 8 male students, with an average age of 22 years.

### 3.2 Research design

#### Testing materials.

To ensure greater reliability and validity, we conducted a pilot test to select the appropriate poems for the current study. Additionally, we considered the lexical complexity and sentence structure of the poems during the selection process. Consequently, we chose relatively less-known seven-character quatrains as the testing material; these poems do not appear in students’ textbooks or well-known poetry anthologies to minimize the influence of students’ prior knowledge on the memory process. Furthermore, during the testing phase, participants were instructed to skip any poems they were already familiar with (if applicable) to ensure that they had not encountered all the selected poems prior to the test. Regarding the difficulty level of these poems, we selected those that presented challenges without excessive complexity, taking into account the language proficiency and literary background of the participants. This approach aims to balance the difficulty level of the poems with the degree of memorization achievable. To achieve this, we conducted a pilot test to determine the appropriate difficulty level of the selected poems [26]. For specific testing material, please refer to S1 Appendix.

#### Testing procedure.

Since memory is the foundation of all learning, students must refrain from interference when answering questions based on their experiences with the test materials. They are required to perform transfer tests that rely solely on their memory as much as possible. Therefore, a transfer test can only be considered valid after the completion of reciting the classical Chinese poems. If the poems have not been recited, the transfer test is deemed invalid and will not be included in the statistical analysis. For example, if a student recited only the first ancient poem but answered questions about both the first and second classical Chinese poems in the transfer test, the response to the second ancient poem would not be included in the statistics. The student’s transfer-level test was conducted on an answer sheet that did not include any schema or the original text of the classical Chinese poems, but rather consisted of a series of questions.

The instructors of the experiment are two of the authors, who have obtained the certificate of teaching and memory competition. The instructors taught students the new method for learning ancient Chinese poetry. Students received training through lectures and group work. The training materials consisted of poems accompanied by poetry schemas, as illustrated in Fig 1. Generally, this experiment lasted for eight school weeks. The control group did not receive any intervention, while the experimental group underwent schema training once a week for the first six weeks, with each session lasting three hours. The content covered schema theory, poem-schema memory exercises, and the application of poem schemas in teaching. Additionally, students in the experimental group were trained to identify keywords, build emotional associations,and utilize visual schemas in the process of learning poems to enhance the transfer under the guidance of the two theories. In the seventh week, both the experimental and control groups took the first test, which consisted of 12 classical Chinese poems that the students had not previously recited. The learning materials for the control group included only the titles, authors, and original texts of the poems, while the materials for the experimental group also incorporated schemas (as shown in Fig 1). The learning period was 10 minutes, during which students were required to study the materials sequentially, and could only progress to the next poem after fully memorizing and understanding the current one. Once all learning was completed, an immediate transfer test (poetry appreciation) was conducted, with a time limit of 10 minutes. The transfer questions (see S2 Appendix), primarily based on the definitions of classical Chinese poetry appreciation outlined in the examination guidelines, were designed from four aspects: content, language, techniques, and emotions, and were scored according to the expected key points. Answer closely aligned with the key points could earn 1 point, and the final score amounted was considered the transfer test score.

Seven days later, in the eighth week, the second transfer test was conducted, which involved a poetry appreciation assessment. The test included questions based directly on the classical Chinese poems studied in the seventh week, with a time limit of 10 minutes and scoring rules similar to those used in the previous assessment [26]. It is important to note that, after the seventh-week test, the learning materials were immediately collected, and the students were not informed that a second test would take place the following week. This approach aimed to minimize the potential interference of review during the interval, thereby enhancing the validity of transfer stability test, which is recommended in previous literature [27].

When the students completed the second test in the eighth week, ten students from the experimental group were randomly selected (using simple random sampling) for an interview to reflect on the learning process and evaluate the effectiveness of the schema method in facilitating the transfer of classical Chinese poetry knowledge.

### 3.3 Data collection and analysis

After completing the two tests, the scores from the poetry transfer tests for both groups were statistically analyzed. The impact of cognitive schemas on transfer ability and stability in learning classical Chinese poetry was assessed by comparing the results of the transfer tests between the two group. The test data were processed using Excel. Following a normality test, it was found that the data for the control group did not follow a normal distribution (p < 0.05). Consequently, the Mann-Whitney U test was employed. The effective size was indicated by the Rank-Biserial Correlation using the formula: r = 1 – (2U)/ (n_1_ × n_2_).

The calculation of transfer ability and stability are as follows: a) the scoring points of transfer ability (i,e., 1 point per item, based on four dimensions: content, language, techniques, and emotions, with a total score cap of 27 points); b) the exact formula used to compute transfer stability (e.g., stability = second test score/first test score × 100%; e.g., if a student scored 10 on the first test and 7 on the second, the transfer stability would be 70%).

The interview data underwent content analysis, which summarized how students utilize cognitive schemas to memorize classical Chinese poems and their attitudes toward this method. Additionally, the learners’ cognitive transfer processes were better understood.

## 4. Results and discussion

### 4.1 Cognitive schema and classical Chinese poetry transfer ability

As shown in Table 1, there is a significant difference (p < 0.05, effective size = 0.856, effective size = 0.896) in the scores between the control group and the experimental group in the two transfer tests. The experimental group achieved higher minimum, maximum, and mean scores on the transfer tests compared to the control group. Therefore, there is a positive effect of cognitive schema on the transfer ability of classical Chinese poetry. Students in the experimental group were better able to complete the poetry transfer test after memorizing the poems. In terms of the minimum scores, the control group recorded a score of 0 in both the first and second transfer tests because participants were unable to memorize any single poem, rendering them incapable of answering the transfer test questions. In contrast, the minimum scores for students in the experimental group ranged from 0 to 4. Although the maximum scores for the control group in the two transfer tests reached 10 to 11 points, the maximum scores for the experimental group approached the full score. The maximum number of classical Chinese poems that participants from both groups could completely memorize within 10 minutes was 6 (from the first poem to sixth one), which corresponds to a score of 15 points (see the supporting information for detail). The difference in mean scores between the two groups is noteworthy. The mean scores for the control group in the first and second transfer tests were 2.81 and 0.97, respectively, indicating a statistically significant decrease according to the Wilcoxon signed-rank test (Z = −5.826, *p* < 0.01). On the contrary, the mean scores for the experimental group in the two transfer tests were 7.76 and 5.29, respectively, significantly surpassing those of the control group (Z = −8.357, *p* < 0.01, *r* = 0.856 for the first test; Z = −8.846, *p* < 0.01, *r* = 0.896 for the first test). These results generally align with previous studies, showing schemas such as visual images or text-image integration or emotions have a positive impact on learners’ transfer ability in learning classical Chinese poetry [28–30].

**Table 1:** Impact of cognitive schema on the transfer ability.

		Min	Max	M	SD	Z	*p*	*r*
The first test	control group	0	11	2.81	2.33	−8.357	.000	.856
	experimental group	4	14	7.76	2.54			
The second test	control group	0	7	0.97	1.48	−8.846	.000	.896
	experiment group	0	12	5.29	2.17			

As illustrated in Table 1, cognitive schemas can significantly enhance learners’ transfer abilities in learning classical Chinese poetry. They enable students to perform better on transfer tests within a limited timeframe. This improvement occurs because the students in the experimental group have already learned and familiarized themselves with the cognitive schemas, which form a cognitive structure in their minds. Therefore, they are able to activate existing knowledge when faced with the transfer test [15], and simultaneously reinforce their previous cognitive structure [16]. This consolidates the interaction between new material and existing cognitive structure, ultimately improving their transfer abilities [17]. For instance, during the interview, all ten participants indicated that cognitive schemas play a significant role in helping them establish connections with existing knowledge, thereby facilitating positive transfer. Additionally, cognitive schemas can assist learners in constructing scenarios through association, mobilizing their emotions, enabling them able to engage with classical Chinese poetry in an “immersive’‘ manner, and fostering greater empathy with the poets [2,3,30,31]. As a result, a closer and more robust association develops between new material and the learners’ existing cognitive experiences and knowledge, which can enhance the transfer of cognitive structures, and improve their transfer abilities. The third interviewee recalled the transfer process: “*When I saw the first two poems, I first thought of all kinds of frontier fortress poems or verses like ‘ the yellow river rises to the white clouds’, ‘beyond the Gate of Jade no vernal wind will blow*’‘. By activating learners’ existing cognitive schemas and further stimulating their interest [32], connections can be established among these schemas, thereby enhancing the learners’ transfer abilities. Additionally, learners can also integrate their real-life experiences [5,23], or even relevant movie scenes to promote the construction and automation of cognitive schemas, and facilitate transfer [33]. For instance, the first interviewee recalled: “*I will associate familiar characters with Chinese costume dramas so that the scene is not easy to forget and the relatedness can be much tighter*.’‘

However, cognitive schemas are characterized by variability, structure, and activity [34], and cognitive structures also exhibit individual differences [16]. Consequently, significant disparities among learners emerge during the transfer process, ultimately resulting in variations in their transfer abilities. For instance, the score range of the experimental group is between 0 and 14 points, with the standard deviation of the experimental group being slightly larger than that of the control group. This observation further highlights the differences in learners’ self-regulation of learning [35]. In the process of learning classical Chinese poetry through cognitive schemas, it is essential to leverage subjective initiative and strategies involving emotions, keywords, and images to connect with established cognitive structures and facilitate positive transfer.

### 4.2 Cognitive schema and stability of classical Chinese poetry transfer

The influence of cognitive schemas on the stability of classical Chinese poetry transfer is reflected in the ratio of scores from the second transfer test compared to those from the first test. According to Table 2, the mean ratio of scores for the experimental group was significantly higher than that of the control group (69.88% > 29.51% Z = −5.619, *p* < 0.01, *r* = 0.570), suggesting that the stability of classical Chinese poetry transfer was greater in the experimental group. Furthermore, the standard deviation for the control group was higher than that of the experimental group, indicating that the ratio of scores in the second transfer test to those in the first test appeared more variable within the control group. This variability suggests that the students in the control group exhibited lower transfer stability, while the relatively consistent ratio in the experimental group indicates a higher level of transfer stability among its students. These results are consistent with previous studies, demonstrating the impact of schemas on the transfer stability of learning classical Chinese poetry [28,30].

**Table 2:** Impact of cognitive schema on the transfer stability.

	Mean ratio	Standard deviation	Z	*p*	*r*
control group	29.51%	38.22%	−5.619	.000	.570
experimental group	69.88%	21.62%			

As evidenced by the data presented above, cognitive schemas can significantly enhance the transfer stability of students learning classical Chinese poetry. This enhancement occurs because cognitive schemas assist students in strengthening their retention of poetry-related memory content, which facilitates their ability to transfer knowledge in poetry learning. This finding suggests that learning classical Chinese poetry through cognitive schemas constitutes meaningful learning, as it could establish non-arbitrary and substantive connections between new material and the learners’ existing cognitive structures. This process leads to cognitive assimilation and the migration of cognitive structures[15], thereby improving both memory retention and transfer stability. Furthermore, learners can only achieve effective transfer and enhance transfer stability by establishing relevant connections with their original cognitive structures, which must be stable and clear [36].

Furthermore, since the students in the experimental group have received training in learning classical Chinese poetry using cognitive schemas, it could be said that advance organizers have bridged the gap between learners’ existing cognitive structures and new material. This approach has strengthened memory retention through “ideational scaffolding’‘ [16,37]. Meanwhile, with the assistance of “advance organizers’‘, learners can enhance the availability and discriminability of their cognitive structures, which promotes positive transfer [9,15–17] and improves transfer stability. The learners’ current cognitive schemas are also interconnected with their prior schemas, facilitating cognitive assimilation [24] and fostering cognitive transfer [25], which contributes to transfer stability.

In summary, the experimental group outperformed the control group in both tests, indicating that learning classical Chinese poetry through cognitive schema can effectively enhance students’ transfer abilities and the stability of their learning processes. This poetry-learning method helps learners connect their learning of new material with their existing cognitive structures by employing strategies such as associations, imagery, emotions, colors, keywords, and the integration of poetry with visual art, as shown in Section 2 and illustrated by Fig 1. Additionally, it regulates related cognitive loads, which can facilitate the automation of schemas and foster positive cognitive transfer. Consequently, this approach improves students’ transferabilities and promotes the stability of their learning transfer.

## 5. Conclusions

This study employs a method that combines controlled experiments and interviews to investigate the impact of cognitive schema on learners’ transfer ability and transfer stability in the context of classical Chinese poetry learning. The results indicate that the experimental group outperformed the control group in both transfer ability and transfer stability. This finding suggests that cognitive schema can enhance positive cognitive transfer among learners, thereby improving their transfer ability and stability in the study of classical Chinese poetry.

Learning classical Chinese poetry can stimulate and cultivate students’ passion for fine traditional Chinese culture while enhancing their cultural literacy. Therefore, both the teaching and learning of classical Chinese poetry should place significant emphasis on the accumulation and recitation of poems. Additionally, the role of advance organizers should be strengthened by connecting the classical Chinese poems that students need to memorize with their existing cognitive structures. This approach builds bridges between cognitive schemas, thereby promoting positive cognitive transfer in the learning of classical Chinese poetry.

It must be acknowledged that this study has several limitations, including a small number of experimental participants, a brief testing duration of only 10 minutes, and the absence of a questionnaire survey to assess the transfer process among all participants. It is recommended that future research should aim to increase the number of participants and extend the testing duration to facilitate further validation. Additionally, both future research and teaching practices should consider further exploration of individual differences, for example, the age, gender and learning styles, in schema use and learning preferences. Finally, further exploration is necessary to optimize the alignment between cognitive schemas and the original poetic concepts of classical Chinese poetry, which necessitates incorporating dimensions such as schema quality, complexity, or activation frequency to deepen our understanding of how internal schema attributes may differentially influence transfer ability and transfer stability.

## Supporting information

S1 File Testing material. (RAR)
